# Burnout and resilience during the COVID-19 outbreak: differences between male and female students

**DOI:** 10.1016/j.heliyon.2022.e10019

**Published:** 2022-07-20

**Authors:** M. Arli Rusandi, Ledya Oktavia Liza, Dominikus David Biondi Situmorang

**Affiliations:** a Riau University, Indonesia; b Lancang Kuning University, Indonesia; c Atma Jaya Catholic University of Indonesia, Indonesia

**Keywords:** Burnout, Resilience, Students, Male, Female, COVID-19

## Abstract

This research aims to determine the burnout levels of male and female students on the three subscales of Exhaustion (EX), Cynicism (CY), and Professional Efficacy (PE) and their resilience during the COVID-19 pandemic. This is a non-experimental quantitative, descriptive, and cross-sectional research conducted on students of Riau University. The snowball sampling method was used to obtain data from 131 students consisting of 69 female and 62 male through the distribution of an online questionnaire and analyzed using the independent sample t-test and Pearson Product Moment Correlation. The data distribution showed that burnout on the PE, EX, CY, and resilience is in the high, moderate, and low categories, respectively.

The data analysis showed that the burnout variable for the PE and EX indicators in female was higher than for men. Meanwhile, male's CY and resilience indicators were higher than female. This indicates significant differences in burnout and resilience between male and female during the pandemic. The results also showed that there is no correlation between PE and resilience as well as CY and resilience, while EX and resilience show a correlation. This finding shows the psychological condition of students in using distance learning during the pandemic. There is a need for strategies to be implemented to reduce the impact of the pandemic on students for better mental health.

## 1 Introduction

Currently, the world is focused on ways to handle the complexity of the COVID-19 pandemic, which has led to the implementation of various policies to break the chain of the spread. Physical distancing is one of the options adopted by many countries to prevent the spread of this virus (Situmorang, 2020). This policy had a significant impact on various sectors, one of which was education, where the government made a sudden decision to conduct online learning, causing many problems (Mamahit and Situmorang, 2022). The problems associated with online learning include stress, anxiety, and depression among students (Fauziyyah et al., 2021; Lindasari et al., 2021).

The introduction of distance learning at the beginning of the COVID-19 pandemic is a new normal for Indonesian students (Handayani et al., 2020). This is because the research of home policy promoted many educators to bombard students with assignments, thereby increasing their pressure and stress levels (Sofyananjani et al., 2021). At first, they might be able to deal with it, but as time goes on, their self-restraint unknowingly becomes weakened. It puts pressure on new students as well as those trying to complete their thesis. This means that due to this pandemic, some students experience pressure that leads to stress (Situmorang, 2021b).

The compensatory response to spontaneous stress is no longer effective in keeping students safe and in harmony. When compensation lasts too long, the stress finally finds its saturation point, which may often cause various symptoms not understood by the person concerned, and this is known as burnout.

According to Maslach et al. (2010), burnout has Exhaustion, Cynicism, and Professional Efficacy indicators. Exhaustion is characterized by fatigue caused by the depletion of emotional energy. It is associated with feelings of boredom, sadness, depression, anxiety, burdened by academic activities, etc. Cynicism refers to a cynical or cold attitude, which makes one stay away from tasks and people, thereby leading to unproductivity. Cynicism is often shown by indifference, reluctance, and laziness to learn. Meanwhile, Professional Efficacy refers to a feeling of self-competence and a desire to excel academically.

Preliminary research on burnout during the pandemic focused more on subjects of medical personnel such as doctors and nurses (Dimitriu et al., 2020; Fessell and Cherniss, 2020; Launer, 2020; Wu et al., 2020). The research by Karakose et al. (2021) mapped research trends related to COVID-19 using subjects dominated by health workers, doctors, and patients, but failed to highlight students' burnout.

Due to the COVID-19 pandemic, which made the government encourage students to keep studying from home, resilience is highly needed (Masten and Motti-Stefanidi, 2020). Reivich and Shatté (2002) reported that resilience is the ability to cope and adapt to severe events or major problems that occur in life, such as emotion regulation, impulse control, optimism, causal analysis, empathy, self-efficacy, reaching out, etc.

According to Turner (2014), individuals with a high level of resilience tend to be less susceptible to stress and remain in excellent condition in completing their tasks. In addition, students with high resilience tend to show a positive attitude in dealing with obstacles. This is also in line with Arce et al. (2009) that individuals with this attribute show positive emotions in dealing with various events. Werner and Smith (1992) stated that the human ability to survive and not give up in difficult life situations is called the concept of resilience. Amelia et al. (2014) concluded that the factors influencing this attribute are stress on campus and adaptation to a new environment.

These factors physically and mentally determine students' success in relating and interacting with the environment, which are basic factors in achieving life and happiness. Furthermore, Wang (2008) stated that resilience is a key variable related to adaptation to the university environment, and those with high levels of this attribute are less prone to stress and stay in top shape at work.

The insights obtained from burnout and resilience research among college students can empower the academic community and develop higher education strategies to welcome the technology-based industrial revolution 5.0. Therefore, from the above background, this research aims to deeply examine burnout and students' resilience during the pandemic by providing answers to the following questions.

1. What was the burnout level of students during the pandemic in the Exhaustion, Cynicism, and Professional Efficacy subscales?
2. What is the level of student resilience during the pandemic?
3. What is the comparison between male and female burnout and resilience during the pandemic?
4. What is the relationship between burnout in the subscale Exhaustion, Cynicism, and Professional Efficacy with student resilience during the pandemic?

## 2 Methods

### 2.1 Participants and procedures

This is a non-experimental quantitative, descriptive, cross-sectional survey research. It aims to determine the description, comparison of resilience and burnout levels between males and females on PE, EX, and CY subscales during the pandemic systematically and factually.

This research was conducted on a total of 131 first-year and final year students of Guidance and Counseling, Riau University, consisting of 69 female and 62 male determined using the snowball sampling technique. Before conducting this research, students were informed to obtain approval for their voluntary participation. Data were collected by distributing online questionnaires designed using a google form link to students for seven days. The results were sent back to their respective emails for confidential purposes. This study has been reviewed by the Ethics Committee of the Department of Guidance and Counseling, Riau University. They decided that the study has been in accordance with ethical standards of Psychology/Guidance and Counseling discipline and Research Ethics Code of Riau University. Furthermore, informed consent was obtained from all participants for this study.

### 2.2 Data collection and analysis

#### 2.2.1 Measures

##### 2.2.1.1 Maslach Burnout Inventory-Student Survey (MBI-SS)

Two scales were used as a data collection tool. The first is the Maslach Burnout Inventory-Student Survey (MBI-SS), originally developed by Chirkowska-Smolak and Kleka (2011) for the subject of the General Survey. This tool was further developed by Schaufeli et al. (2002) to measure burnout in students specifically. It consists of three subscales, namely Exhaustion (EX), Cynicism (CY), and Professional Efficacy (PE), with 16 question items. Points on this scale include sentences such as *"if you have never felt this way, write the number "0" (zero) in the place before the statement,"* and *"if you have had this feeling, indicate with the numbers 1 to 6.”* On this scale, equivalence is carried out through a back-translation process, expert validation, and instrument testing. According to Maslach et al. (2010), the scores of three burnout dimensions cannot be combined to determine burnout because the MBI is a measure of three independent constructs. Instrument testing was carried out on 100 randomly selected students, and the results were declared valid (rxy: 0.264–0.641), with an alpha coefficient of 0.723.

##### 2.2.1.2 Brief Resilience Scale (BRS)

The BRS developed by Smith et al. (2008), which contains six items that measure the ability to bounce back from stress, was also used to determine students' resilience. Scale points were chosen between 1 (strongly disagree) and 5 (strongly agree). The equivalence is determined by carrying out a back-translation process, expert validation, and instrument testing on 100 randomly selected students. All items were declared valid in the instrument test results (rxy: 0.436–0.734), with an alpha coefficient of 0.752. BRS Score Interpretation of 1.00–3.00, 3.01–4.30, and 4.31–5.00 indicates Low, Normal, and High resilience, respectively.

### 2.3 Main analyses

The data collected were analyzed using the SPSS 26 statistical software package with an independent t-test to determine whether there were significant differences between male and female students on the MBI-SS and BRS items. Furthermore, Pearson Product Moment Correlation analysis was used to determine the relationship between burnout and resilience. Before analyzing the data, to evaluate whether the data contributed normally, the One-Sample Kolmogorov-Smirnov Test was conducted.

## 3 Results and discussion

The calculation results from 131 student samples obtained an average of 3 burnout dimensions, namely the Professional Efficacy (PE), Exhaustion (EX), and Cynicism (CY), in the high, moderate, and low categories, with average scores of 25.59, 15.13 and 9.2. Furthermore, the distribution of data for the resilience level of 131 students was obtained in the Normal category with an average score of 3.21.

After conducting the One-Sample Kolmogorov-Smirnov test, the Asymp value was performed with a Sig. (2-tailed) of 0.2 greater than 0.05. This shows that the data to be measured contributes normally. Based on this, an independent sample t-test analysis can be conducted to determine whether there is a significant difference between male and female students and Pearson Product Moment Correlation analysis to determine the relationship between burnout and resilience.

**Figure 1:**
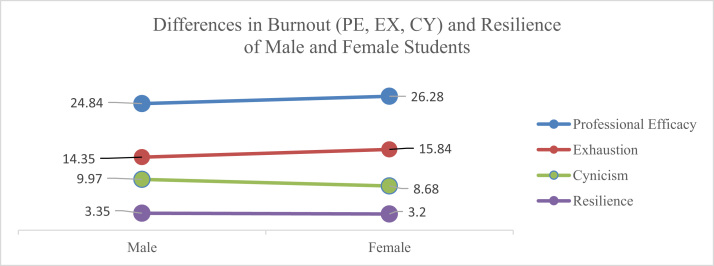
Differences in burnout and resilience of male and female students.

A t-test was also conducted to determine the score obtained and evaluate the difference in the scores of male and female students. The t-test was used to determine whether the scores obtained from the scale used in this research differed according to the sex variable, and the results are shown in Table 1 and Figure 1.

**Table 1:** Statistical description and descriptive testing of differences in burnout and resilience of male and female students.

Instrument	Item Group	n	Mean Score (SDa)	Sig. (2-tailed)	t	Mean dif.d	95% CIe Cohen's		Cohen's D
							Lower	Upper	
MBI	Professional Efficacy	M = 62	24.84	0.304	-1.032	-1.437	-4.192	1.318	0.435
		F = 69	26.28						
	Exhaustion	M = 62	14.35	0.234	-1.195	-1.486	-3.946	0.975	0.634
		F = 69	15.84						
	*Cynicism*	M = 62	9.97	0.139	1.488	1.287	-0.424	2.997	0.430
		F = 69	8.68						
BRS		M = 62	3.35	0.179	1.350	0.152	-0.071	0.375	0.432
		F = 69	3.20

The results of data analysis using different tests Independent Sample T-test showed that female have higher Professional Efficacy than male at 26.28 vs. 24.84, with a t value of -1.032 > 0.05, hence, there is a significant difference. In the Exhaustion group, female were also in the higher category than male with 15.84 vs. 14.35, at a t value of -1.195 > 0.05, therefore, there is a significant difference. Meanwhile, in the Cynicism group item, male were slightly higher than female with 9.97 vs. 8.68, at a t value of 1.488 > 0.05, indicating there was a significant difference. In BRS, male were in the higher category than female with 3.35 vs. 3.20, at a t values of 0.179 > 0.05, indicating a significant difference.

Pearson Product Moment Correlation analysis was further conducted to determine the relationship between burnout in the subscales, namely Exhaustion (EX), Cynicism (CY), and Professional Efficacy (PE) with resilience. The result showed that there is no correlation between PE and resilience as well as CY and resilience at scores of r = 0.095, p = 0.280 and r = -0.167, p = 0.057, respectively. While the EX and resilience scores of r = -0.253 and p = 0.004 indicates a correlation.

The calculation results of the three dimensions of burnout show that the Exhaustion (EX) and Cynicism (CY) are in the moderate category, which indicates that students experience burnout during distance learning. However, the dimension of Professional Efficacy (PE) is in the High category, which can be an asset for students to rise from burnout.

Smith et al. (2008) stated that there is a relationship between resilience and optimism, social support, and active coping, enabling students to adapt to burnout difficulties during online learning. Optimism is one of the keys to resilience because optimistic people have a favorable attitude towards an event with a calm mental state (Carver et al., 2010; Kelberer et al., 2018).

Social support is a fairly broad concept, encompassing all matters concerning the significance of the existence of others to family, friends, and co-workers (Verheijden et al., 2005). According to Wilks and Spivey (2009) and Woodhead et al. (2016), it is indispensable in managing and reducing stress.

Active coping is the perception that one must be able to master and overcome psychological and environmental pressures with hard work and earnest determination (Stevens-Watkins et al., 2014). Based on preliminary research, male students are more likely to have lower Professional Efficacy and Exhaustion. Professional Efficacy is a feeling of competence and achievement in academic work, and an example of an item on this scale is *“In my opinion, I am a good student.”* Furthermore, Exhaustion is a depletion of emotional energy, in contrast to the physical or mental process. An example of an item on this scale is *“I feel emotionally drained by my studies.”* Meanwhile, male Cynicism, which is one emotional, social and cognitive connection with their academic work, fellow students, and others on campus, tends to be higher. An example of an item on this scale is *“I doubt the importance of my studies.”* This multivariate analysis yielded significant interactions between male and female.

This is different from Worly et al. (2019) that male students are likely to have lower Exhaustion and higher Professional Efficacy scores (MBI-SS). The transient fatigue and stress scores were statistically significant. Other research also indicated that female showed higher levels of global fatigue and emotional/physical exhaustion, while male reported lower levels of decreased sense of accomplishment (Armon et al., 2010).

Maslach et al. (2001) stated that most studies or literature provided inconsistent analysis regarding sex differences in burnout. Furthermore, Purvanova & Muros (2010) concluded that female tend to feel more exhausted, but male report higher depersonalization. Other research also reported that female are more likely to have lower burnout than male (Hildebrandt et al., 2012).

These research showed mixed results, which can be related to the environment, background, and how each individual lives. This is in line with the research conducted by Dilullo et al. (2011) that millennials have a variety of backgrounds, personalities, learning, and thinking styles (Situmorang and Salim, 2021). Therefore, they have technological and multitasking tendencies, with the ability to read, think, and produce professional behavior. These attributes show that students in the millennials may not be homogeneous regarding learning strategies and attitudes. The results of this research relate to the current COVID-19 outbreak, where the activities of each individual are no longer what they used to be, with the emergence of various mental reactions and adjustments.

Arnout et al. (2020) stated that the adaptation process during the pandemic can trigger mental health symptoms such as anxiety, depression, stress, etc. Furthermore, the research conducted by Dedovic et al. (2009) stated that the socialization process plays a very important role in promoting social dependence on female and independence related to a sense of competence in men. Based on this, it can be concluded that gender differences do not affect an individual burnout, specifically during the current pandemic.

Research conducted for resilience showed that male have a higher insignificant resilience rate than female. This was also found in the research conducted by Sarwar et al. (2010) that male have a higher resilience than female, although it is not related to their academic achievement. Shen et al. (2022) also stated that societal resilience occurs with changes in lifestyle, education level, and socio-demographic characteristics.

Based on this description, it can be concluded that the COVID-19 outbreak has different impacts on the mental health of each individual, as well as their economy, environment, and background. Research also showed that individuals respond differently to emotional distress caused by traumatic events, similar with the current pandemic (Killgore et al., 2020). Interventions that can be conducted to overcome burnout and resilience problems use a solution-focused brief counseling approach, which has proven effective in increasing self-efficacy (Rusandi, 2017), persistence (Rusandi et al., 2019) and self-esteem (Rusandi and Rachman, 2014). In addition, new findings are currently being developed regarding *"rapid counseling/psychotherapy"* to cure various mental health problems in the COVID-19 era (Situmorang, 2021a; 2021c).

## 4 Conclusion

The results revealed that the student burnout from the Professional Efficacy (PE), Exhaustion (EX), and Cynicism (CY) dimensions, as well as the resilience, are in the high, moderate, moderate, and normal categories. The data analysis result showed significant differences between female and male. Furthermore, this research showed no correlation between PE and resilience and CY and resilience, as opposed to EX and resilience. This showed that it is important to provide intervention to students to minimize the negative effects of the pandemic.

The numerous negative effects of the COVID-19 pandemic experienced by students affect their mental health, interfering with their courses. Therefore, campuses should pay more attention to students' mental health to minimize the associated negative effects.

## Declarations

### Author contribution statement

M. Arli Rusandi: Conceived and designed the experiments; performed the experiments; Wrote the paper.

Ledya Oktavia Liza: Analyzed and interpreted the data; Contributed reagents, materials, analysis tools or data; Wrote the paper.

Dominikus David Biondi Situmorang: Contributed reagents, materials, analysis tools or data; Wrote the paper.

### Funding statement

This research did not receive any specific grant from funding agencies in the public, commercial, or not-for-profit sectors.

### Data availability statement

Data included in article/supp. material/referenced in article.

### Declaration of interests statement

The authors declare no conflict of interest.

### Additional information

No additional information is available for this paper.

## Appendix A Supplementary data

Maslach Burnout Inventory-Student Survey _spl_MBI-SS_spl__ Maslach Burnout Inventory-Student Survey _spl_MBI-SS_spl__

BRS BRS
