# Supplementary material for: Burnout and resilience during the COVID-19 outbreak: differences between male and female students
Source: Heliyon. 2022 Jul 20;8(8):e10019. doi: 10.1016/j.heliyon.2022.e10019 (PMC9297693; doi:10.1016/j.heliyon.2022.e10019)
Supplement: Maslach Burnout Inventory-Student Survey _spl_MBI-SS_spl__ [file mmc1.docx]

**Maslach Burnout Inventory-Student Survey (MBI-SS)**

**Instructions**: On the following page are 16 statements of job-related feelings. Please read each statement carefully and decide if you ever feel this way about your job. If you have never had this feeling, write the number "0" (zero) in the space before the statement. If you have had this feeling, indicate how often you feel it by writing the number (from 1 to 6) that best describes how frequently you feel that way. An example is shown below.

**How often:**

0= Never

1= A few times a year or less

2= Once a month or less

3= A few times a month

4= Once a week

5= A few times a week

6= Every Day

1. I feel bored with my online class activities
2. I have become less interested in learning activities since I enrolled at the University of Riau
3. I can effectively solve the problems that arise in my studies
4. I feel exhausted during the last hour of online class
5. I become less enthusiastic about starting learning activities
6. I believe if I can do well in the class, I'm taking
7. I feel the burden of studying on campus is too heavy
8. In my opinion, I am an optimistic student in dealing with lecture assignments
9. I have learned a lot of exciting things in the courses on campus
10. I feel tired when I have to get up early and go to campus/do an online class
11. I think the lessons in online classes are useless
12. I feel excited when the learning outcomes I achieve are satisfactory
13. Studying in online classes is very burdensome for me
14. I doubt the importance of studying for myself
15. While in online classes, I believe that if I can concentrate my time studying
16. When I'm in an online class or studying, I don't want to be bothered by anyone
