# Supplementary material for: Burnout and resilience during the COVID-19 outbreak: differences between male and female students
Source: Heliyon. 2022 Jul 20;8(8):e10019. doi: 10.1016/j.heliyon.2022.e10019 (PMC9297693; doi:10.1016/j.heliyon.2022.e10019)
Supplement: BRS [file mmc2.docx]

Brief Resilience Scale (BRS)

| **Respond to each statement below by circling one answer per row.** | | **Strongly Disagree** | **Disagree** | **Neutral** | **Agree** | **Strongly Agree** |
| --- | --- | --- | --- | --- | --- | --- |
| **BRS 1** | I tend to bounce back quickly after hard times. | 1 | 2 | 3 | 4 | 5 |
| **BRS 2** | I have a hard time making it through stressful events. | 5 | 4 | 3 | 2 | 1 |
| **BRS 3** | It does not take me long to recover from a stressful event. | 1 | 2 | 3 | 4 | 5 |
| **BRS 4** | It is hard for me to snap back when something bad happens. | 5 | 4 | 3 | 2 | 1 |
| **BRS 5** | I usually come through difficult times with little trouble. | 1 | 2 | 3 | 4 | 5 |
| **BRS 6** | I tend to take a long time to get over setbacks in my life. | 5 | 4 | 3 | 2 | 1 |

**Scoring:** Add the value (1-5) of your responses for all six items, creating a range from 6-30. Divide the sum by the total number of questions answered (6) for your final score.

**Total score:** / 6

**My score:** (average)
